# Vascular Loop of the Anterior Inferior Cerebellar Artery (AICA) as a Cause of Sensorineural Hearing Loss (SNHL): A Case Report

**DOI:** 10.7759/cureus.42838

**Published:** 2023-08-02

**Authors:** Corinne A O'Brien, Nithin Gupta, Varun Kasula, Meredith Lamb, Richard Alexander

**Affiliations:** 1 Otolaryngology, Campbell University School of Osteopathic Medicine, Lillington, USA; 2 Otolaryngology, Granville Ear, Nose, and Throat, Oxford, USA

**Keywords:** internal auditory canal, vestibulocochlear nerve, sensorineural hearing loss, tinnitus, anterior inferior cerebellar artery, vascular loop

## Abstract

Sensorineural hearing loss (SNHL) is one of the most common causes of hearing loss worldwide. Although highly prevalent, many patients often present with SNHL of unknown cause. Related to SNHL is tinnitus, which often presents with SNHL and can have debilitating effects on patients. The idiopathic nature of SNHL and tinnitus often makes treatment difficult, however, a relatively new etiology has been suggested as a cause of SNHL and tinnitus -- vascular loops within the internal auditory canal (IAC). This report presents the case of a 36-year-old male with bilateral SNHL and tinnitus treated with oral steroids. The patient reported subjective improvement of hearing loss and tinnitus, and the audiogram demonstrated hearing improvement, except in higher frequencies. After initial treatment, MRI revealed a vascular loop of the anterior inferior cerebral artery (AICA) in the right IAC, in contact with the vestibulocochlear nerve. Thus, this case report seeks to present a conservative strategy for SNHL and tinnitus in the presence of a vascular loop of the AICA. As a controversial cause of SNHL and tinnitus, there is no standard of treatment for AICA loops of the IAC which are often treated surgically. This case highlights the importance of an initial conservative prior to surgical intervention. Thus, we seek to contribute to the growing body of literature by further elucidating the relationship between SNHL, tinnitus, and vascular loops and discussing potential pathophysiological mechanisms to guide optimal management strategies.

## Introduction

The World Health Organization estimates that by 2050, the incidence of hearing loss will approach approximately 2.5 billion with 700 million people requiring intervention [1]. The most common cause of hearing loss is sensorineural hearing loss (SNHL), encompassing any pathology of the cochlea, vestibulocochlear nerve, or auditory processing in the brain. Common etiologies of SNHL include congenital abnormalities, ototoxicity, meningitis, diabetes, and noise-induced hearing loss [2]. Interestingly, although highly prevalent, an estimated 71% of patients with SNHL are classified as idiopathic due to no identifiable etiology [3]. 

Associated, though not correlated, with SNHL is tinnitus -- defined by the Mayo Clinic as “ringing in the ears even though no external sound is present” and includes phantom noises characterized as buzzing, hissing, or clicking [4]. Tinnitus, particularly in those who experience it chronically, can become an increasingly debilitating condition. Individuals suffering from severe tinnitus reportedly experience higher rates of anxiety, depression, and insomnia than individuals without tinnitus [5-7]. There is a strong association between the incidence of hearing loss and tinnitus which has led many to believe that improving one’s hearing can decrease tinnitus or mask one’s perception of his or her tinnitus [8]. In individuals with both hearing loss and tinnitus, treatment may be complicated due to the often-idiopathic nature of hearing loss. 

Recently, there have been reports in the literature of vascular loops such as the anterior inferior cerebellar artery (AICA) within the internal auditory canal (IAC) and/or cerebellopontine angle (CPA), in both normal and symptomatic individuals. Although tinnitus, vertigo, and hearing loss are observed in many diseases, in rare cases, these may also be a manifestation of the neurovascular conflict between the AICA and vestibulocochlear nerve [9]. This study seeks to present the case of a patient presenting with tinnitus/hearing loss and an AICA loop within the IAC while reviewing possible pathophysiological mechanisms for these findings. 

## Case presentation

**Figure 1:**
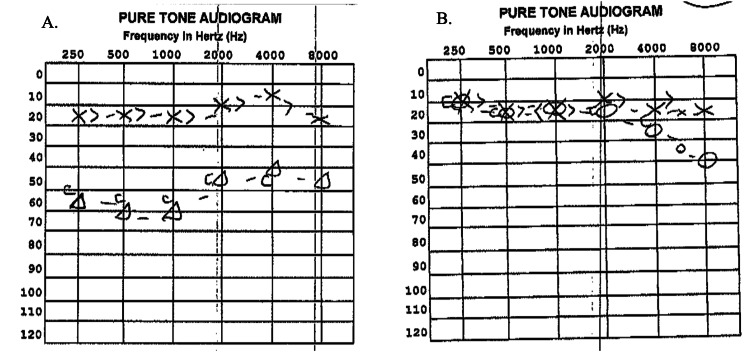
A. Results of pure tone audiometry pre-oral steroid treatment. B. Results of pure tone audiometry post steroid treatment demonstrate SNHL in the highest frequencies with improvement. SNHL, sensorineural hearing loss

A 36-year-old male with a history of supraventricular tachycardia, ascending aorta dilation, and obesity presented to urgent care with a one-month history of sudden onset right-sided non-pulsatile tinnitus (described as “hissing"), and room-spinning vertigo. He was treated with meclizine and augmentin. His tinnitus persisted, and he presented to the emergency room for his continued tinnitus and intermittent vertigo. His work-up was reassuring, and he was discharged home with an Otolaryngologist. The patient presented to our office approximately five weeks after the onset of his tinnitus and vertigo. An audiogram was obtained (Figure 1A) which revealed type A tympanogram bilaterally and moderate SNHL across all frequencies tested. A presumed diagnosis of sudden SNHL was made, and the patient was treated with a high dose, of prednisone taper for 20 days. The patient returned with subjectively improved hearing loss and improved tinnitus. His repeat audiogram revealed mild sloping to moderate SNHL on the right from 6000 to 8000 Hz (Figure 1B).

**Figure 2:**
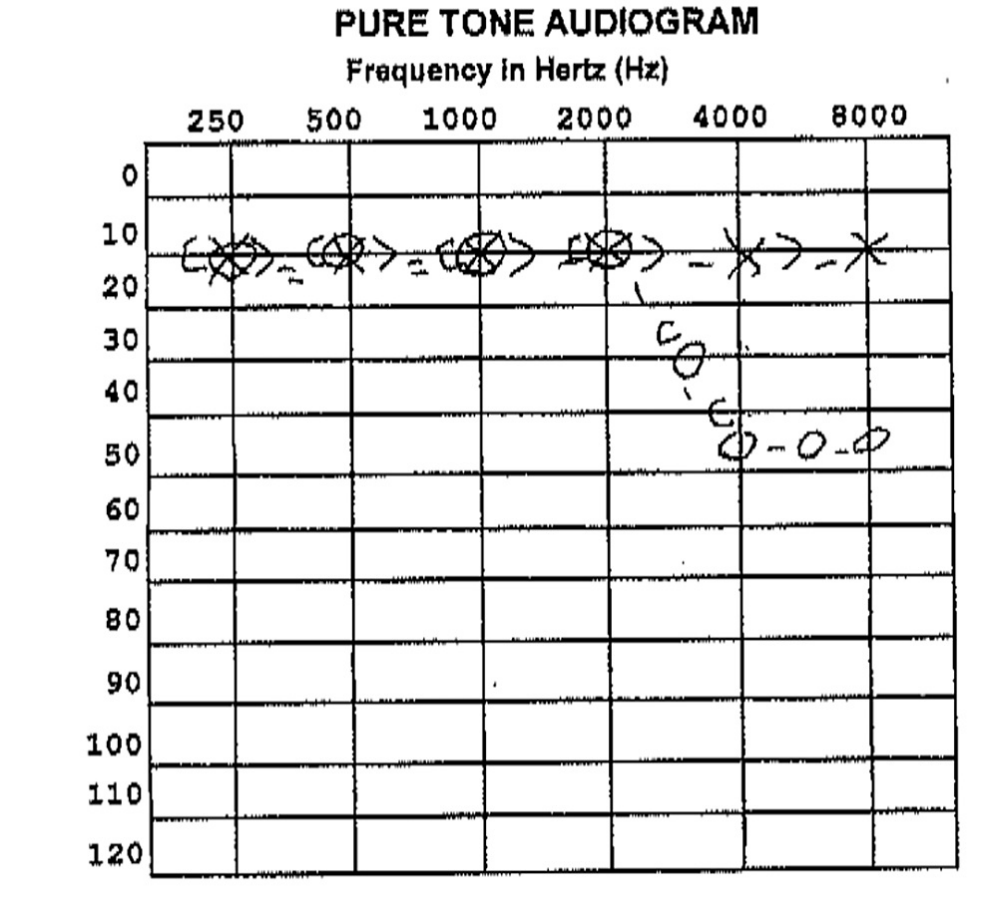
Results of pure tone audiometry demonstrates SNHL in the highest frequencies; slightly worse than the previous audiogram. SNHL, sensorineural hearing loss

Intratympanic steroids were discussed, but the patient declined. An MRI was ordered, but the patient could not tolerate it due to claustrophobia. Auditory brainstem response (ABR) testing was performed which showed normal responses bilaterally. A third audiogram revealed mild sloping to moderate SNHL from 3000 to 8000 Hz in the right ear (Figure 2). 

**Figure 3:**
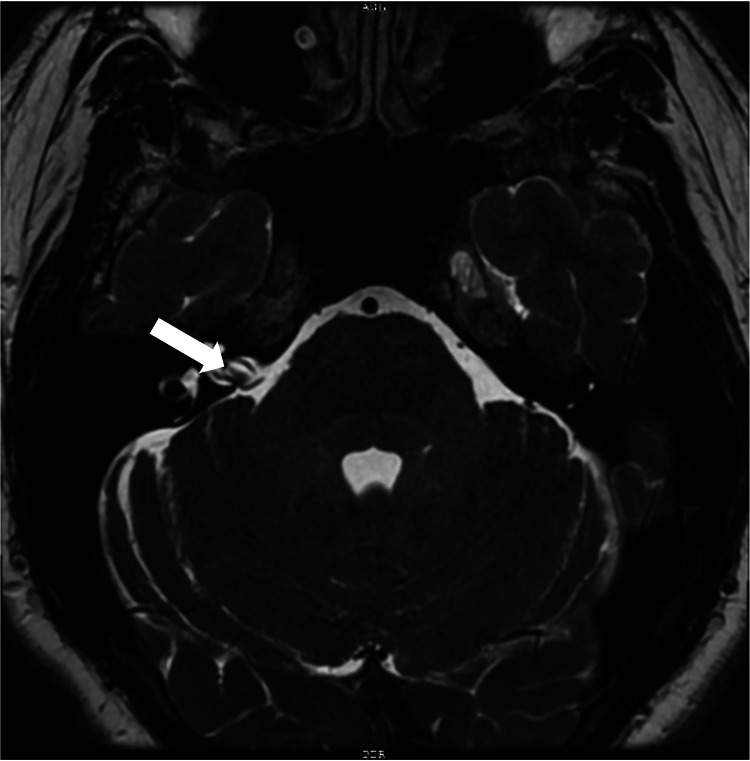
Multiplanar brain MRI with IV contrast indicates AICA loop against the vestibulocochlear nerve with no compression (white arrow). AICA, anterior inferior cerebral artery

The limitations of the ABR study were explained to the patient, and he elected to proceed with an open-bore MRI with attention to the IAC. The MRI revealed a prominent vascular loop of the anterior inferior cerebellar artery (AICA) within the IAC (Figure 3).

Radiological examination demonstrated no restriction diffusion, abnormal enhancement of the vestibulocochlear nerve, or any mass effect. Further discussions with our radiology colleagues confirmed that the loop was directly against the vestibulocochlear nerve without compression and that the AICA loop filled approximately 75% of the IAC. The results were discussed with the patient, and a referral for surgical management was offered. The patient elected for a conservative approach and will return for audiometric testing in six months. 

## Discussion

In this report, we present the case of a patient with tinnitus, sudden SNHL, and a vascular loop of the AICA within the IAC. SNHL itself is a very common presentation, however, in most patients, there is no identifiable cause [2]. One of the most controversial etiologies is the presence of vascular loops within the IAC. In 2007, a blinded analysis of 167 MRI scans of the CPA demonstrated 94% of patients had an AICA loop of varying classifications. Among those patients, 66 were diagnosed with unexplained unilateral hearing loss [10]. Similarly, a 2016 study found that of 536 patients that underwent surgical treatment-microvascular decompression (MVD)-for neurovascular compression of the vestibulocochlear nerve by the AICA, tinnitus was reported in only nine patients and SNHL in one patient [9]. Conversely, Chadha and Weiner demonstrated through a systematic review and meta-analysis of observational studies that there was a significant association between vascular loops in contact with the vestibulocochlear nerve and SNHL, but not non-pulsatile tinnitus, similar to the patient presented in this case [11]. Interestingly, they reported subjects with pulsatile tinnitus are 80 times more likely to have vascular loops in contact with the AICA, compared to non-pulsatile [11]. This is in contrast to studies which report no association between the type of tinnitus and hearing loss or type of AICA loop and subtype of tinnitus [12]. Thus, the complex relationship between the subtype of tinnitus and AICA loop contact of the vestibulocochlear nerve must be further elucidated, including in patients who describe tinnitus outside of the classical subtypes.

Attempts have been made to elucidate the exact pathophysiology of SNHL/tinnitus caused by vascular loops, with theories including direct compression of the nerve by the loop, demyelination of the nerve due to contact with the artery, and reduced perfusion leading to hypoxia of the nerve [13-14]. In the present study, no obvious compression of the vestibulocochlear nerve indicates that the symptoms may have been caused by hypoxia or micro compression. Interestingly, treatment with steroids improved the patient’s tinnitus and hearing loss except in the higher frequencies. Although the first-line treatment for sudden SNHL is steroids to reduce inflammation, it is plausible in this case that steroid treatment may have resolved the acute cause of the patient’s symptoms which unmasked the primary cause of the underlying SNHL caused by the AICA loop. Furthermore, as treatment in our patient improved only to a degree, hypoxic damage or demyelination could have occurred to the hair cells in a high-frequency region of the cochlea which would not be repaired with steroid administration even if the vascular loop was the primary cause of the patient’s hearing loss.

With such uncertainty regarding not only the association of vascular loops with SNHL/tinnitus but also the exact pathophysiology involved, there remains no standardized scaling system to guide treatment when vascular loops are suspected. A lack of uniform grading systems for vascular loops can also cause confusion. This is exemplified in another study that found that tinnitus was associated with the configuration of the AICA when using the Kazawa grading system, but not the Chavda or Gorrie classification [15]. Additionally, even with standardized grading systems, there is potential for inter-observer differences which may impact meta-analyses seeking to find associations between SNHL and vascular loops [14]. Thus, there is a need for a global grading system to determine the possible impact/association of vascular loops on tinnitus and SNHL to help guide treatment. Currently, the standard of treatment reported in the literature is MVD, which often involves moving the vascular loop away from the nerve via a retrosigmoid approach [9]. In a case series involving 12 patients with AICA compression, MVD was used to treat hearing loss and tinnitus with an improvement of 5 dB or more in seven patients [16]. In cases such as the one presented in this report; the patient received steroid treatment which was shown by a pure tone audiogram to improve hearing to a degree. Although this is the case for a single patient, our data suggest that treatment with steroids may be the first line in patients with hearing loss and a vascular loop within the IAC. If no improvement is noticed, MVD could be offered. Further investigation should seek to understand possible hypoxia-induced damage to the cochlea caused by vascular loops and treatment modalities focused on this mechanism. 

Although there have been studies that have found no correlation between the vascular loops and SNHL/tinnitus, there have been reports which have shown MVD of the vascular loop completely or partially resolving symptoms of tinnitus, vertigo, or SNHL [9, 17-20]. Furthermore, the establishment of a normalized grading scale will allow physicians to understand the pathology and determine whether to start with surgical or medical treatment. Finally, with the advent of advanced imaging techniques and physiologically accurate in vitro models such as organoids, steps should be taken to thoroughly examine the proposed mechanisms of SNHL caused by vascular loops.

## Conclusions

We report the case of a vascular loop of the AICA in contact with the vestibulocochlear nerve, as a possible cause of SNHL and tinnitus. With a lack of standardized guidelines for treatment, it is important for providers to consider offering nonsurgical options to patients prior to surgery. As the relationship between SNHL/tinnitus and vascular loops remain unclear, large-scale studies are needed to confidently demonstrate the relationships between these. Further, steps must be taken to further investigate mechanisms for pathophysiology through advanced imaging techniques and in vitro models.
